# Assessing the 9G Technology Blood Test for Predicting Lung Cancer in Patients with CT-Detected Lung Nodules: A Multicenter Clinical Trial

**DOI:** 10.3390/cancers16223737

**Published:** 2024-11-05

**Authors:** So Yeon Kim, Young Sik Park, In Ae Kim, Hee Joung Kim, Kye Young Lee

**Affiliations:** 1 Division of Pulmonary and Critical Care Medicine, Seoul National University Hospital, Seoul 03080, Republic of Korea; lydia0000@hanmail.net (S.Y.K.); mdyspark@gmail.com (Y.S.P.); 2 Precision Medicine Lung Cancer Center, Konkuk University Medical Center, Seoul 05029, Republic of Korea; iakim@kuh.ac.kr (I.A.K.); hjkim@kuh.ac.kr (H.J.K.)

**Keywords:** lung nodule, chest CT, biomarker, 9G technology, lung cancer

## Abstract

**Background and Objectives**: Lung nodules detected by chest computed tomography (CT) often require invasive biopsies for definitive diagnosis, leading to unnecessary procedures for benign lesions. A blood-based biomarker test that predicts lung cancer risk in CT-detected nodules could help stratify patients and direct invasive diagnostics toward high-risk individuals. **Methods**: In this multicenter, single-blinded clinical trial, we evaluated a test measuring plasma levels of p53, anti-p53 autoantibodies, CYFRA 21-1, and anti-CYFRA 21-1 autoantibodies in patients with CT-detected lung nodules. Sensitivity, specificity, positive predictive value (PPV), and negative predictive value (NPV) were calculated, and subgroup analyses by gender, age, and smoking status were performed. A total of 1132 patients who had CT-detected lung nodules, including 885 lung cancer cases and 247 benign lesions, were enrolled from two academic hospitals in South Korea. **Results**: The test demonstrated a sensitivity of 78.4% (95% CI: 75.7–81.1) and specificity of 93.1% (95% CI: 90.0–96.3) in predicting lung cancer in CT-detected nodules. The PPV was 97.6%, and the NPV was 54.6%. Performance was consistent across gender (sensitivity 79.3% in men and 76.8% in women) and age groups, with a specificity of 93.4% in men and 92.7% in women. Stage I lung cancer was detected with a sensitivity of 80.6%. **Conclusions**: The Lung Cancer test based on 9G technology presented here offers a non-invasive method for stratifying lung cancer risk in patients with CT-detected nodules. Its integration into clinical practice could reduce unnecessary interventions and foster earlier detection.

## Simple Summary

Lung nodules detected by computed tomography (CT) often require invasive procedures for definitive diagnosis. With the increasing use of CT, incidental lung nodules have increased significantly. An adjunctive blood-based biomarker test that predicts lung cancer risk could reduce unnecessary interventions and focus diagnostic efforts on high-risk patients. This study introduces a blood-based biomarker test that predicts lung cancer risk in CT-detected nodules with a sensitivity of 78.4% (95% CI: 75.7–81.1) and a specificity of 93.1% (95% CI: 90.0–96.3).

## 1. Introduction

Lung cancer remains the leading cause of cancer-related deaths worldwide [1,2,3]. Many patients are diagnosed at advanced stages, worsening the challenge of effective treatment and resulting in a meager 5-year survival rate [4,5]. Patients diagnosed at early stages (stage 1) exhibit markedly higher survival rates compared to those diagnosed in the locally advanced and metastasized stages (54.8% vs. 27.4% vs. 4.2%, respectively) [6,7]. This underscores the critical importance of lung cancer diagnosis at stage 1 in a population-based screening to significantly improve patient outcomes and save lives. Lung cancer screening initiatives targeting stage 1 hold immense potential for reducing mortality and increasing the 5-year survival rates associated with lung cancer [8].

National Lung Screening Trials (NLSTs) have documented that implementing low-dose computed tomography (LDCT) in lung cancer screening can lower lung cancer mortality by approximately 20% [9,10]. The screening studies have underscored various drawbacks linked to the LDCT, such as invasive work-up and the treatment of benign nodules and morbidity [11,12]. The distinction between low-risk and high-risk lung cancer patients is necessary for nodules detected on low-dose chest CT as they may experience more harm than benefit [12,13]. The Serial LDCT-based Lung Computed Tomography Screening Reporting and Data System (Lung-RADS) has been recommended in many studies to standardize the reporting and management of screening findings [14]. The Lung-RADS lacks the accuracy to discriminate between benign nodules and early-stage malignant cancer, leading to a high rate of false-positive results (96.4%) [15]. Targeted approaches that allow the identification of individuals at high risk of lung cancer after LDCT screening can reduce the overall burden and focus the treatment efforts on these individuals [16].

A Lung Cancer test based on 9G technology [17] is a lateral flow strip membrane test that detects and measures the CYFRA 21-1, CYFRA 21-1-Anti-CYFRA 21-1 autoantibody complex, p53, and p53-Anti-p53 autoantibody complex [18,19]. The autoantibodies show 5–10 times higher levels in cancer patients than in healthy individuals [20,21,22] and can be detected in blood up to 3–4 years before symptomatic presentation in patients with solid tumors [23,24]. In a previously reported clinical study spanning 244 lung cancer patients and 359 healthy individuals, the Lung Cancer test has shown a specificity of 97.3% and a sensitivity of 75.0% [18]. This paper reports the results of a multicenter, single-blinded, retrospective, clinical performance study (*n* = 1399) to evaluate the performance of the Lung Cancer test to classify patients with a confirmed nodule on chest CT findings into high-risk or low-risk categories of lung cancer.

## 2. Materials and Methods

### 2.1. Study Design

This multicenter, single-blinded study evaluated the performance of a blood-based diagnostic test for predicting lung cancer risk in patients with CT-detected lung nodules. The primary objective was to determine the test’s sensitivity in detecting lung cancer by measuring plasma levels of cancer proliferation markers (p53, p53-anti-p53 autoantibody complex) and lung cancer-specific markers (CYFRA 21-1, CYFRA 21-1-anti-CYFRA 21-1 autoantibody complex). Secondary objectives included determining the positive and negative predictive values and evaluating the test’s performance across different stages and histological classifications.

Data from in vitro diagnostic devices and multi-marker tests show that sensitivities typically range from 37% to 77%, averaging 62.2%, and specificities between 80% and 91%, averaging 84.7% [25,26,27,28]. Based on these benchmarks, the minimum performance criteria for this test were set at 62% sensitivity and 85% specificity. A previous study reported a sensitivity of 75.0% and specificity of 97.3% [18]. For this trial, the target performance was set at 69% sensitivity (minimum 62%) and 92% specificity (minimum 85%), with a significance level of 2.5% and statistical power of 98%. To achieve these targets, the required sample size was calculated using Equations (S1) and (S2). The calculated sample sizes for lung cancer cases and benign cases were 704 and 196, respectively. A 20% buffer for dropout was included, resulting in a final recruitment target of 1050 lung cancer cases and 349 benign cases.

The study was conducted at two academic hospitals in South Korea, including Seoul National University Hospital and Konkuk University Hospital. The study protocols were approved by the institutional review boards at Seoul National University Hospital (No. D-2210-098-1370) and Konkuk University Hospital (No. 2022-10-061-003). Archived samples with written informed consent for future research use were used.

### 2.2. Study Population

Patients aged 50 to 79 years with CT-detected lung nodules between 2009 and 2020 were eligible for inclusion. Patients with unanalyzable blood samples or insufficient clinical data were excluded. AJCC cancer staging manual (8th version) was followed for staging of NSCLC samples [29].

### 2.3. Laboratory Methods

#### 2.3.1. Blood Sample Storage and Processing Method

The whole blood sample was collected into an anticoagulant tube and treated with EDTA (lavender-topped). Samples were then centrifuged for 10 min in refrigerated centrifuge at 1000–2000× *g* to separate blood cells from plasma (platelet depletion can occur if the centrifugation at 2000× *g* extends for 15 min). Samples were then separated into 0.5 mL aliquots and stored at 2–8 °C for immediate use or at −20 °C or below for later use. Multiple freeze–thaw cycles were avoided to retain the validity of test results. Samples that are hemolyzed, lipemic, or icteric were not used as they may impact the test results. Blood samples were randomly assigned into two groups, with each group analyzed by independent teams blinded to clinical information during this research.

#### 2.3.2. Diagnostic Test

The 9G test^TM^ Cancer/Lung test (Biometrix Technology Inc., Chuncheon, Republic of Korea) was used to detect the lung cancer-related biomarkers, including p53 (CS-2), p53-anti-p53 autoantibody complex (CS-1), CYFRA 21-1 (LS-2), and CYFRA 21-1-anti-CYFRA 21-1 autoantibody complex (LS-1) by following the manufacturer’s protocol. A 20 µL of plasma from each patient was introduced into four separate reaction tubes containing reagents specific to each biomarker. The biomarkers were detected through biocomplex formation between target molecules in the plasma and monoclonal antibodies linked to Fluorobeads (FDs) and Latexbeads (LDs).

For CS-1 detection, the reaction involved Fluorobeads conjugated with monoclonal human IgG and Latexbeads conjugated with monoclonal p53 capture antibody, which formed a biocomplex p53-anti-p53 autoantibody complex with p53 in the plasma. CS-2 detection involved the reaction between Fluorobeads conjugated with monoclonal p53 detection antibody and Latexbeads conjugated with p53 capture antibody, which forms a biocomplex with free p53 present in the plasma. For LS-1 detection, the reaction targeted CYFRA 21-1-anti-CYFRA 21-1 autoantibody complex using Fluorobeads conjugated with human IgG and Latexbeads conjugated with monoclonal CYFRA 21-1 capture antibody. LS-2 detection involved the reaction between Fluorobeads conjugated with monoclonal CYFRA 21-1 detection antibody and Latexbeads conjugated with CYFRA 21-1 capture antibody, which forms a biocomplex with free CYFRA 21-1 protein in the plasma.

Each reaction was processed by the automated 9G-1000 system, which loads the samples, performs the washing steps with a buffer solution, and automatically calculates the signal for each marker (Supporting Information Link S1 for video guide). After the reactions, the respective values CS-1, CS-2, LS-1, and LS2 were recorded and used for the determination of an Index value using Equation (S3).

### 2.4. Statistical Analysis

Continuous variables were reported as mean ± standard deviation (SD), and categorical variables as frequencies and percentages. Test performance was assessed by calculating sensitivity, specificity, positive predictive value (PPV), and negative predictive value (NPV), all with 95% confidence intervals (CIs). Statistical comparisons were performed using the chi-square test for categorical variables, Fisher’s exact test when appropriate, and Student’s *t*-test or Wilcoxon rank-sum test for continuous variables. Statistical analysis was performed using MedCalc version 17.4.4 (MedCalc Software, Mariakerke, Belgium).

## 3. Results

**Figure 1:**
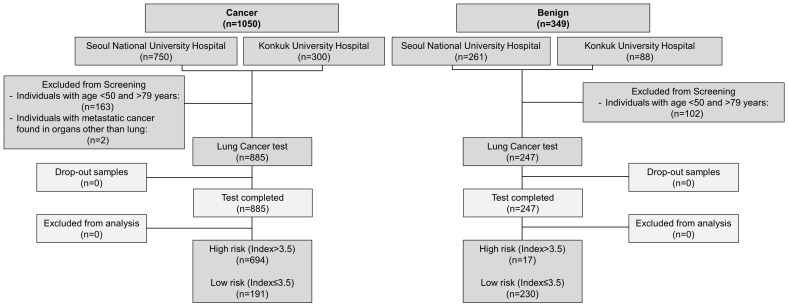
A flow chart indicates the recruitment of samples for evaluating the clinical performance of the Lung Cancer test.

A total of 1132 patients (885 lung cancer and 247 benign) were included (Figure 1). A total of 1399 samples comprising 1050 cancer and 349 benign samples were initially recruited for this study. A total of 2/1050 cancer samples that showed metastasis to other organs and 163/1050 cancer samples from individuals with age < 50 and >79 were excluded from this study. Similarly, 102/349 benign samples were excluded from this study based on age criteria (<50 Age > 79). A total of 1132 remaining samples (Cancer, *n* = 885; benign, *n* = 247) were then tested using the Lung Cancer test. All samples were discreetly analyzed by blinding the information of the origin and types of the samples.

There were 885 lung cancer cases and 247 benign cases in our study. Table 1 and Table S1 show the characteristics of samples included in this study. Sample specimens were collected from about 703 (62.1%) men and 429 (37.9%) women. The average age of patients with lung cancer and with benign diagnosis were 67.0 years (range: 50.0–79.0 years) and 66.7 years (range: 50.0–79.0 years), respectively. Smoking history was checked in both cancer patients and patients with benign diseases. Among the 885 cancer cases, 62.8% were identified as smokers, and 37.2% of patients never smoked. In patients with benign disease, 50.6% of cases were identified as smokers, and 49.4% of patients never smoked. Among the 885 cancer patients, 757 (85.5%) and 128 (14.5%) patients were diagnosed with non-small cell lung cancer (NSCLC) and small cell lung cancer (SCLC), respectively. Among the 757 NSCLC cases, lung cancer stages were identified as Stage I (408, 53.9%), Stage II (142, 18.8%), Stage III (146, 19.3%), and Stage IV (61, 8.1%). The 128 SCLC cases were identified as limited disease (57, 44.5%) and extensive disease (71, 55.5%).

**Figure 2:**
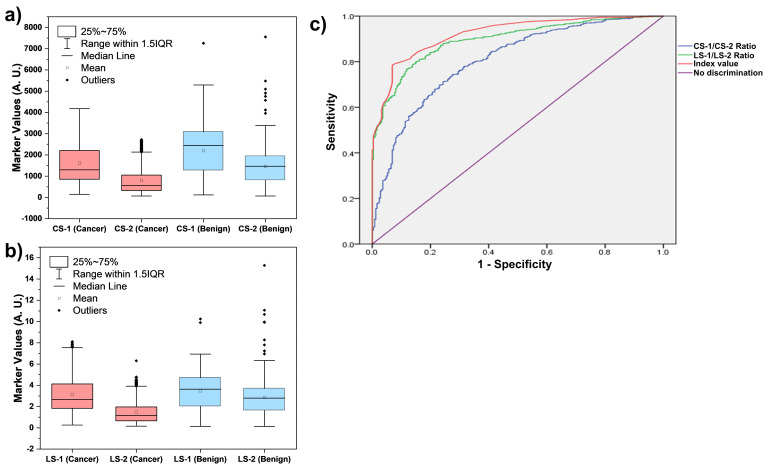
Graph representing the raw values of (**a**) CS-1 (p53-anti-p53 autoantibody complex) and CS-2 (p53), (**b**) LS-1 (CYFRA 21-1-anti-CYFRA 21-1 autoantibody complex) and LS-2 (CYFRA21-1) in cancer and benign patient samples. (**c**) ROC curve analyses comparing two ratios CS-1/CS-2, LS-1/LS-2, and the Index value for distinguishing lung cancer from benign cases.

All 1132 samples were tested discreetly using the Lung Cancer test to determine the levels of CS-1, CS-2, LS-1, and LS-2, as shown in Figure 2a,b. It is clear from Figure 2 that the levels of these four markers alone do not allow efficient discrimination between lung cancer and benign patients. However, as shown in Figure 2c, an ROC curve of the Index values calculated using the levels of these four biomarkers can efficiently differentiate between lung cancer and benign patients. The ROC analyses of the two ratios (CS-1/CS-2 and LS-1/LS-2) and the Index values are presented in Figure 2c. The cut-off Index value of 3.5 was determined at the area under the curve (AUC) of 0.928. Thus, an Index value of 3.5 was selected to discriminate the patients with a high-risk (Index > 3.5) and a low-risk (Index ≤ 3.5) of lung cancer. The cut-off of 3.5 achieved a sensitivity of 78.4% (95% CI: 75.7–81.1) and a specificity of 93.1% (95% CI: 90.0–96.3) for diagnosing all lung cancer stages (I–IV). These results are in accordance with the results in a recent report, where a cut-off of 3.60 achieved 81.0% sensitivity and 95.0% specificity for diagnosing all LC stages (I–IV) [30].

As shown in Table 2 (Figure S1), about 694 (78.4%) cancer patients were identified as high-risk for lung cancer, and 191 (21.6%) cancer patients were identified as low-risk for lung cancer. Based on these results, the sensitivity and specificity of the Lung Cancer test for lung cancer diagnosis were 78.4% and 93.1%, respectively. The positive and negative predictive values were found to be 97.6% and 54.6%, respectively, with an overall concordance value of 81.6%.

The obtained data were also analyzed to determine the gender-specific sensitivity and specificity of the Lung Cancer test to identify the risk of lung cancer. As shown in Table S2 (Figure S2), out of 566 men, about 449 (79.3%) cancer patients were identified as high-risk for lung cancer, and 117 (20.7%) cancer patients were identified as low-risk for lung cancer. Out of 319 women, about 245 (76.8%) cancer patients were identified as high-risk for lung cancer, and 74 (23.2%) cancer patients were identified as low-risk for lung cancer. Thus, at a specificity of 93.4%, the sensitivity of the Lung Cancer test for the identification of men at a high risk of lung cancer was found to be 79.3%. Similarly, the Lung Cancer test demonstrated 76.8% sensitivity and 92.7% specificity for identifying women at a high risk of lung cancer.

As shown in Table S3 (Figure S3), the ability of the Lung Cancer test for age-specific identification of the risk of lung cancer was evaluated. The Lung Cancer test demonstrated sensitivity (specificity) values of 78.4% (94.7%), 79.5% (93.1%), and 77.8% (92.0%) for the identification of individuals at a high risk of lung cancer in the age groups of 50–59 years, 60–69 years, and 70–79 years, respectively. Smoking history-specific sensitivity (specificity) of the Lung Cancer test for identification of individuals at a high risk of lung cancer among smokers and non-smokers was found to be 78.8% (94.4%) and 77.8% (91.8%), respectively, as shown in Table S4 (Figure S4).

As mentioned earlier, 757/885 cancer patients were diagnosed with NSCLC, whereas, 128/885 (14.5%) cancer patients were diagnosed with SCLC. We analyzed the Index values in these two categories to estimate the sensitivity and specificity of the Lung Cancer test for histology-specific identification of individuals at a high risk of lung cancer. As shown in Table S5 (Figure S5), the tumor tissue histology-based sensitivity and specificity of the Lung Cancer test for identification of high-risk lung cancer patients were 78.1% and 93.1% for patients with NSCLC. In SCLC patients, the sensitivity and specificity of the Lung Cancer test were 80.5% and 93.1%.

As shown in Table S6 (Figure S6), the Index cut-off value of 3.5 allows the Lung Cancer test to identify patients at a high risk of cancer at stages I, II, III, IV, LD, and ED with sensitivities of 80.6%, 73.2%, 74.0%, 82.0%, 82.5%, and 78.9%, respectively, at a specificity of 93.1%.

## 4. Discussion

Even though lung cancer screening plays a crucial role in detecting lung cancer at multiple stages (Stage I–II), the prevalence of false positives, which lead to overdiagnosis, remains a significant concern in cancer screening programs [31,32]. Traditionally, chest X-rays (CXRs) and sputum cytology have been the standard procedures for screening lung tumors. Yet, these traditional methods fail to identify lung cancer effectively during its initial stages [33,34]. On the contrary, LDCT has emerged as a highly effective method for mass screening lung cancer, offering a significant improvement in detecting early-stage lung tumors [35]. The Early Lung Cancer Action Project (ELCAP) findings indicated that LDCT scans were six-fold more precise and sensitive than CXRs [36]. The utilization of LDCT in large-scale screenings has been instrumental in identifying lung cancers at stage 1, enhancing the five-year survival rates among patients [37,38]. Clinical trials have confirmed that LDCT-based screening can lead to a 20% decrease in lung cancer mortality and a 7% reduction in overall mortality compared to CXRs, establishing LDCT as the superior method currently available for lung cancer screening [10,39].

Despite its advantages, the major limitation of LDCT lies in its high rate of false positives, with 96% of positive results being non-cancerous upon further investigation through biopsy [39]. These facts underscore the need for a test to complement LDCT and help distinguish between true and false positives. While biopsy remains the definitive method for cancer diagnosis, it is impractical to perform on a large scale due to the sheer volume of positive LDCT results. Therefore, a non-invasive blood-based biomarker test is greatly sought to accompany LDCT in lung cancer screening [21,40,41]. The Lung Cancer test, a non-invasive blood-based biomarker test, can enhance the diagnostic process and significantly reduce the rate of false positives associated with LDCT [42,43,44], making it a valuable tool in the early detection and management of lung cancer.

The results of our multicenter, single-blinded clinical performance study provide compelling evidence for the efficacy of the Lung Cancer test in identifying individuals at a high risk of lung cancer. The study’s findings are particularly significant given the urgent need for effective screening tools to distinguish between low-risk individuals and those who may benefit most from further diagnostic evaluation. The Lung Cancer test presented here demonstrated a sensitivity of 78.4% and a specificity of 93.1%, indicating a high degree of accuracy in detecting lung cancer among patients with confirmed nodules in chest CT findings. These performance metrics are strengthened by the positive predictive value of 97.6% and a negative predictive value of 54.6%, underscoring its potential as a valuable adjunct to LDCT screening in community settings.

The gender-specific analysis revealed that the test maintains a high specificity of 93.4% in men and 92.7% in women, with sensitivities of 79.3% and 76.8%, respectively. These results suggest that the Lung Cancer test is equally effective across genders, which is crucial for its application in a diverse population. The age-specific evaluations of the Lung Cancer test further support its robustness, with the sensitivity and specificity remaining consistent across different age groups. This consistency is essential for the in vitro diagnostic test and its utility in population-based screening programs, which often encompass a wide age range.

The Lung Cancer test demonstrated the ability to identify the patients that are at a high risk of cancer irrespective of histological cancer types, including NSCLC and SCLC, indicated by the sensitivities of 78.1% and 80.5%, respectively, at a specificity of 93.1%. The high sensitivity and specificity of the Lung Cancer test add another layer of precision to its diagnostic capabilities. Moreover, the Index cut-off value of 3.5 effectively stratifies patients into high-risk and low-risk categories, facilitating targeted intervention for those most likely to benefit from it. The Lung Cancer test’s high sensitivity across smoker and non-smoker groups indicates that it can correctly identify patients with a high risk of lung cancer.

The early-stage detection of lung cancer is a critical component in the fight against lung cancer. It involves identifying cancer at stage 1 when it is still localized and has not spread to other parts of the body, which is paramount in improving patient outcomes. The Lung Cancer test allows the detection of NSCLC stage I and stage II lung cancers with a sensitivity of 80.6% and 73.2%, respectively, at a specificity of 93.1%, whereas ED, categorized as SCLC based on histology, was detected with a sensitivity of 82.5% at a specificity of 93.1%.

Notably, the Lung Cancer test facilitated the early-stage detection of both NSCLC and SCLC, with high sensitivity and specificity. The integration of this test into lung cancer screening protocols along with LDCT holds the potential to significantly advance early-stage lung cancer detection and significantly reduce the false positive rate, which are crucial to transform the landscape of lung cancer management.

## 5. Conclusions

In conclusion, the results of our clinical performance study provide compelling evidence for the efficacy of the Lung Cancer test in identifying individuals at a high risk of lung cancer. With a sensitivity of 78.4% and a specificity of 93.1%, the test demonstrates high accuracy in detecting lung cancer. The sensitivities for the detection of NSCLC and SCLC were 78.1% and 80.5%, respectively. The sensitivities for the detection of Stage I, Stage II, Stage III, and Stage IV lung cancers were 80.6%, 73.2%, 74.0%, and 82.0%, respectively, at a specificity of 93.1%. Gender-specific analysis revealed consistent effectiveness across genders, while age-specific evaluations demonstrated consistency across different age groups, which is essential for its application in diverse populations. Therefore, we believe that integrating the Lung Cancer test presented here into lung cancer screening protocols can significantly advance early-stage lung cancer detection and potentially transform the landscape of lung cancer management.

## Figures and Tables

**Table 1:** Characteristics of lung cancer and benign cases analyzed by the Lung Cancer test.

Parameter	Cancer (*n* = 885)	Benign (*n* = 247)	Overall (*n* = 1132)
**Gender, *n* (%)**			
a man	566 (63.9)	137 (55.5)	703 (62.1)
a woman	319 (36.1)	110 (44.5)	429 (37.9)
**Age (years)**			
*n*	885	247	1132
means	67.0 ± 7.35	65.7 ± 8.31	66.7 ± 7.58
median	67.0	66.0	67.0
min~max	50.0–79.0	50.0–79.0	50.0–79.0
**Age category, *n* (%)**			
50~59	153 (17.3)	75 (30.4)	228 (20.1)
60~69	380 (42.9)	72 (29.2)	452 (39.9)
70~79	352 (39.8)	100 (40.5)	452 (39.9)
**Smoking history, *n* (%)**			
Smoker	556 (62.8)	125 (50.6)	681 (60.2)
Never smoker	329 (37.2)	122 (49.4)	451 (39.8)
**Histological classification, *n* (%)**			
Non-Small Cell Lung Cancer (NSCLC)	757 (85.5)	-	757 (85.5)
Small Cell Lung Cancer (SCLC)	128 (14.5)	-	128 (14.5)
Stage of lung cancer—^†^ for NSCLC, *n* (%)			
Stage I	408 (53.9)	-	408 (53.9)
Stage II	142 (18.8)	-	142 (18.8)
Stage III	146 (19.3)	-	146 (19.3)
Stage IV	61 (8.06)	-	61 (8.06)
**Stage of lung cancer—^‡^ for SCLC, *n* (%)**			
Limited Disease (LD)	57 (44.5)	-	57 (44.5)
Extensive Disease (ED)	71 (55.5)	-	71 (55.5)

*n*, number of samples included in the analysis for each group; *n*, number of samples used in the actual analysis for each group; SD, standard deviation; ^†^, AJCC cancer staging manual (8th version) was followed for staging of NSCLC samples; ^‡^, among cancers, SCLC samples were analyzed as the standard (denominator).

**Table 2:** Sensitivity and specificity of Lung Cancer test for identification of risk of lung cancer based on Index value.

Parameter	High Risk of Lung Cancer (Index > 3.5)	Low Risk of Lung Cancer (Index ≤ 3.5)	Sensitivity (%) (95% CI)	Specificity (%) (95% CI)
Cancer (*n* = 885)	694 (78.4%)	191 (21.6%)	78.4 (75.7, 81.1)	93.1 (90.0, 96.3)
Benign (*n* = 247)	17 (6.98%)	230 (93.1%)	-	-

*n*, number of samples included in analysis for each group.

## Data Availability

The data that support the findings of our study are available from the corresponding author upon request.

## Supplementary Materials

The following supporting information can be downloaded at https://www.mdpi.com/article/10.3390/cancers16223737/s1, Equation (S1): Equation for calculating the number of samples required for intended sensitivity of the test; Equation (S2): Equation for calculating the number of samples required for intended specificity of the test; Link S1: 9G test™ Lung Cancer Kit user guide; Equation (S3): Equation for calculation of Index Value; Table S1: Clinical samples (*n* = 1399) collected at Konkuk University Hospital (*n* = 388, 27.7%), and Seoul National University Hospital (1011, 72.3%); Figure S1: Sensitivity, Specificity, PPV and NPV of Lung Cancer test for identification of risk of lung cancer; Table S2: Gender-specific sensitivity and specificity of 9G test^TM^ Cancer/Lung test for identification of risk of lung cancer; Figure S2: Gender-specific sensitivity and specificity of Lung Cancer test for identification of risk of lung cancer; Table S3: Sensitivity and specificity of the 9G test^TM^ Cancer/Lung test for identifying the risk of lung cancer in various age groups; Figure S3: Age group-specific sensitivity and specificity of Lung Cancer test for identification of risk of lung cancer; Table S4: Smoking history-specific sensitivity and specificity of Lung Cancer test for identification of risk of lung cancer; Figure S4: Smoking history-specific sensitivity and specificity of Lung Cancer test for identification of risk of lung cancer; Table S5: Tumor tissue histology-based sensitivity and specificity of Lung Cancer test for identification of risk of lung cancer; Figure S5: Tumor tissue histology-based sensitivity and specificity of Lung Cancer test for identification of risk of lung cancer; Table S6: Lung cancer stage-specific sensitivity and specificity of 9G test^TM^ Cancer/Lung test for identification of risk of lung cancer; Figure S6: Lung cancer stage-specific sensitivity and specificity of Lung Cancer test for identification of risk of lung cancer.

## References

[1] Siegel R.L., Miller K.D., Jemal A. (2018). Cancer statistics, 2018. CA Cancer J. Clin..

[2] Siegel R.L., Miller K.D., Jemal A. (2019). Cancer statistics, 2019. CA Cancer J. Clin..

[3] Siegel R.L., Miller K.D., Wagle N.S., Jemal A. (2023). Cancer statistics, 2023. CA Cancer J. Clin..

[4] Pastorino U., Silva M., Sestini S., Sabia F., Boeri M., Cantarutti A., Sverzellati N., Sozzi G., Corrao G., Marchianò A. (2019). Prolonged lung cancer screening reduced 10-year mortality in the MILD trial: New confirmation of lung cancer screening efficacy. Ann. Oncol..

[5] Oudkerk M., Liu S., Heuvelmans M.A., Walter J.E., Field J.K. (2021). Lung cancer LDCT screening and mortality reduction—Evidence, pitfalls and future perspectives. Nat. Rev. Clin. Oncol..

[6] Wang X., Liu H., Shen Y., Li W., Chen Y., Wang H. (2018). Low-dose computed tomography (LDCT) versus other cancer screenings in early diagnosis of lung cancer: A meta-analysis. Medicine.

[7] Siegel R.L., Giaquinto A.N., Jemal A. (2024). Cancer statistics, 2024. CA Cancer J. Clin..

[8] de Koning H.J., van der Aalst C.M., de Jong P.A., Scholten E.T., Nackaerts K., Heuvelmans M.A., Lammers J.J., Weenink C., Yousaf-Khan U., Horeweg N. (2020). Reduced Lung-Cancer Mortality with Volume CT Screening in a Randomized Trial. N. Engl. J. Med..

[9] National Lung Screening Trial Research T. (2019). Lung Cancer Incidence and Mortality with Extended Follow-up in the National Lung Screening Trial. J. Thorac. Oncol..

[10] National Lung Screening Trial Research T., Aberle D.R., Adams A.M., Berg C.D., Black W.C., Clapp J.D., Fagerstrom R.M., Gareen I.F., Gatsonis C., Marcus P.M. (2011). Reduced lung-cancer mortality with low-dose computed tomographic screening. N. Engl. J. Med..

[11] Baptiste J.V., Jankowich M., Nici L.L. (2017). Lung Cancer Screening with Low Dose CT: Two Year Experience at Providence Veteran Affairs Medical Center. C30. Lung Cancer Screening: Who, Why, Where, and How Much.

[12] Hammer M.M., Byrne S.C., Kong C.Y. (2022). Factors Influencing the False Positive Rate in CT Lung Cancer Screening. Acad. Radiol..

[13] Wu Z., Tan F., Xie Y., Tang W., Wang F., Xu Y., Cao W., Qin C., Dong X., Zheng Y. (2023). A strategy to reduce the false-positive rate after low-dose computed tomography in lung cancer screening: A multicenter prospective cohort study. Cancer Med..

[14] Toumazis I., Erdogan S.A., Bastani M., Leung A., Plevritis S.K. (2021). A Cost-Effectiveness Analysis of Lung Cancer Screening With Low-Dose Computed Tomography and a Diagnostic Biomarker. JNCI Cancer Spectr..

[15] Boiselle P.M. (2013). Computed tomography screening for lung cancer. JAMA.

[16] Oudkerk M., Devaraj A., Vliegenthart R., Henzler T., Prosch H., Heussel C.P., Bastarrika G., Sverzellati N., Mascalchi M., Delorme S. (2017). European position statement on lung cancer screening. Lancet Oncol..

[17] Song K.-S., Balasaheb Nimse S., Kim J., Kim J., Nguyen V.-T., Ta V.-T., Kim T. (2011). 9G DNAChip: Microarray based on the multiple interactions of 9 consecutive guanines. Chem. Commun..

[18] Choe W., Chae J.D., Lee B.-H., Kim S.-H., Park S.Y., Nimse S.B., Kim J., Warkad S.D., Song K.-S., Oh A.-C. (2020). 9G TestTM Cancer/Lung: A Desirable Companion to LDCT for Lung Cancer Screening. Cancers.

[19] Song K.-S., Nimse S.B., Warkad S.D., Oh A.-C., Kim T., Hong Y.J. (2019). Quantification of CYFRA 21-1 and a CYFRA 21-1–anti-CYFRA 21-1 autoantibody immune complex for detection of early stage lung cancer. Chem. Commun..

[20] Tan E.M., Zhang J. (2008). Autoantibodies to tumor-associated antigens: Reporters from the immune system. Immunol. Rev..

[21] Agarwal S., Saini S., Parashar D., Verma A., Sinha A., Jagadish N., Batra A., Suri S., Gupta A., Ansari A.S. (2013). The novel cancer-testis antigen A-kinase anchor protein 4 (AKAP4) is a potential target for immunotherapy of ovarian serous carcinoma. Oncoimmunology.

[22] Zaenker P., Gray E.S., Ziman M.R. (2016). Autoantibody Production in Cancer—The Humoral Immune Response toward Autologous Antigens in Cancer Patients. Autoimmun. Rev..

[23] Patel A.J., Tan T.-M., Richter A.G., Naidu B., Blackburn J.M., Middleton G.W. (2022). A highly predictive autoantibody-based biomarker panel for prognosis in early-stage NSCLC with potential therapeutic implications. Br. J. Cancer.

[24] Li Y., Karjalainen A., Koskinen H., Hemminki K., Vainio H., Shnaidman M., Ying Z., Pukkala E., Brandt-Rauf P.W. (2005). p53 autoantibodies predict subsequent development of cancer. Int. J. Cancer.

[25] Jung Y.J., Oh I.-J., Kim Y., Jung J.H., Seok M., Lee W., Park C.K., Lim J.-H., Kim Y.-C., Kim W.-S. (2018). Clinical Validation of a Protein Biomarker Panel for Non-Small Cell Lung Cancer. J. Korean Med. Sci..

[26] Baldwin D.R., Callister M.E., Crosbie P.A., Dowd E.L., Rintoul R.C., Robbins H.A., Steele R.J.C. (2021). Biomarkers in lung cancer screening: The importance of study design. Eur. Respir. J..

[27] Doseeva V., Colpitts T., Gao G., Woodcock J., Knezevic V. (2015). Performance of a multiplexed dual analyte immunoassay for the early detection of non-small cell lung cancer. J. Transl. Med..

[28] Hajian-Tilaki K. (2014). Sample size estimation in diagnostic test studies of biomedical informatics. J. Biomed. Inform..

[29] Amin M.B., Greene F.L., Edge S.B., Compton C.C., Gershenwald J.E., Brookland R.K., Meyer L., Gress D.M., Byrd D.R., Winchester D.P. (2017). The Eighth Edition AJCC Cancer Staging Manual: Continuing to build a bridge from a population-based to a more “personalized” approach to cancer staging. CA Cancer J. Clin..

[30] Kim H., Lee J.K., Kim H.R., Hong Y.J. (2024). Enhanced Lung Cancer Detection Using a Combined Ratio of Antigen-Autoantibody Immune Complexes against CYFRA 21-1 and p53. Cancers.

[31] González Maldonado S., Motsch E., Trotter A., Kauczor H.-U., Heussel C.-P., Hermann S., Zeissig S.R., Delorme S., Kaaks R. (2021). Overdiagnosis in lung cancer screening: Estimates from the German Lung Cancer Screening Intervention Trial. Int. J. Cancer.

[32] Patz E.F., Pinsky P., Gatsonis C., Sicks J.D., Kramer B.S., Tammemagi M.C., Chiles C., Black W.C., Aberle D.R., Team N.O.M.W. (2014). Overdiagnosis in low-dose computed tomography screening for lung cancer. JAMA Intern. Med..

[33] Oken M.M., Hocking W.G., Kvale P.A., Andriole G.L., Buys S.S., Church T.R., Crawford E.D., Fouad M.N., Isaacs C., Reding D.J. (2011). Screening by chest radiograph and lung cancer mortality: The Prostate, Lung, Colorectal, and Ovarian (PLCO) randomized trial. JAMA.

[34] Marcus P.M., Bergstralh E.J., Zweig M.H., Harris A., Offord K.P., Fontana R.S. (2006). Extended lung cancer incidence follow-up in the Mayo Lung Project and overdiagnosis. J. Natl. Cancer Inst..

[35] Becker N., Motsch E., Trotter A., Heussel C.P., Dienemann H., Schnabel P.A., Kauczor H.U., Maldonado S.G., Miller A.B., Kaaks R. (2020). Lung cancer mortality reduction by LDCT screening-Results from the randomized German LUSI trial. Int. J. Cancer.

[36] Henschke C.I. (2000). Early lung cancer action project: Overall design and findings from baseline screening. Cancer.

[37] Wu F.Z., Kuo P.L., Huang Y.L., Tang E.K., Chen C.S., Wu M.T., Lin Y.P. (2019). Differences in lung cancer characteristics and mortality rate between screened and non-screened cohorts. Sci. Rep..

[38] Henschke C.I., Yip R., Shaham D., Zulueta J.J., Aguayo S.M., Reeves A.P., Jirapatnakul A., Avila R., Moghanaki D., Yankelevitz D.F. (2021). The Regimen of Computed Tomography Screening for Lung Cancer: Lessons Learned Over 25 Years From the International Early Lung Cancer Action Program. J. Thorac. Imaging.

[39] Sozzi G., Boeri M. (2014). Potential biomarkers for lung cancer screening. Transl. Lung Cancer Res..

[40] Seijo L.M., Peled N., Ajona D., Boeri M., Field J.K., Sozzi G., Pio R., Zulueta J.J., Spira A., Massion P.P. (2019). Biomarkers in Lung Cancer Screening: Achievements, Promises, and Challenges. J. Thorac. Oncol..

[41] Bamji-Stocke S., van Berkel V., Miller D.M., Frieboes H.B. (2018). A review of metabolism-associated biomarkers in lung cancer diagnosis and treatment. Metabolomics.

[42] Ostrin E.J., Bantis L.E., Wilson D.O., Patel N., Wang R., Kundnani D., Adams-Haduch J., Dennison J.B., Fahrmann J.F., Chiu H.T. (2021). Contribution of a Blood-Based Protein Biomarker Panel to the Classification of Indeterminate Pulmonary Nodules. J. Thorac. Oncol..

[43] Mazzone P.J., Sears C.R., Arenberg D.A., Gaga M., Gould M.K., Massion P.P., Nair V.S., Powell C.A., Silvestri G.A., Vachani A. (2017). Evaluating Molecular Biomarkers for the Early Detection of Lung Cancer: When Is a Biomarker Ready for Clinical Use? An Official American Thoracic Society Policy Statement. Am. J. Respir. Crit. Care Med..

[44] Paez R., Kammer M.N., Tanner N.T., Shojaee S., Heideman B.E., Peikert T., Balbach M.L., Iams W.T., Ning B., Lenburg M.E. (2023). Update on Biomarkers for the Stratification of Indeterminate Pulmonary Nodules. Chest.

