# Supplementary material for: Assessing the 9G Technology Blood Test for Predicting Lung Cancer in Patients with CT-Detected Lung Nodules: A Multicenter Clinical Trial
Source: Cancers (Basel). 2024 Nov 5;16(22):3737. doi: 10.3390/cancers16223737 (PMC11593157; doi:10.3390/cancers16223737)
Supplement: Supplementary file 1 [file cancers-16-03737-s001.zip › cancers-3271833-supplementary.pdf]

Assessing the 9G Technology Blood Test for Predicting Lung Cancer in Patients with CT-Detected Lung Nodules: A Multicenter Clinical Trial

So Yeon Kim [1], Young Sik Park [1], In Ae Kim [2], Hee Joung Kim [2] and Kye Young Lee [2,*]

[1] Division of Pulmonary and Critical Care Medicine, Seoul National University Hospital, Seoul 03080, Republic of Korea; lydia0000@hanmail.net (S.Y.K.); mdyspark@gmail.com (Y.S.P.)
[2] Precision Medicine Lung Cancer Center, Konkuk University Medical Center, Seoul 05029, Republic of Korea; iakim@kuh.ac.kr (I.A.K.); hjkim@kuh.ac.kr (H.J.K.)
* Correspondence: kyleemd@kuh.ac.kr; Tel.: +82-10-8896-3916

Supporting information

$$n_{Se} = \frac{(Z_\alpha + Z_\beta)^2 \times p_1(1-p_1)}{(p_1-p_0)^2} = \frac{(1.96 + 2.05)^2 \times 0.69(1-0.69)}{(0.69- 0.62)^2} = 703.25 \cong 704 \quad \text{............ (S1)}$$

$$n_{Sp} = \frac{(Z_\alpha + Z_\beta)^2 \times p_1(1-p_1)}{(p_1-p_0)^2} = \frac{(1.96 + 1.64)^2 \times 0.92(1-0.92)}{(0.92- 0.85)^2} = 195.19 \cong 196 \quad \text{............ (S2)}$$

Where, α is the significance level (one-sided test), β is the type 2 error, p_1 is the target performance of test device, and p_1 is the minimum expected performance.

Link S1: 9G test™ Lung Cancer Kit user guide

https://www.youtube.com/watch?v=kbBlS_NpqVI

$$\textbf{Index Value} = \left[\left(\frac{\text{CS}-1}{\text{CS}-2}\right)\left(\frac{\text{LS}-1}{\text{LS}-2)}\right)\right] \text{............(S3)}$$

Where, CS-1 is the value for p53-anti-p53 autoantibody complex, CS-2 is the value for p53, LS-1 is the value for CYFRA 21-1-anti-CYFRA 21-1 autoantibody complex, and LS-2 is the value for CYFRA21-1.

Table S1. Clinical samples (n=1399) collected at Konkuk University Hospital (n=388, 27.7%), and Seoul National University Hospital (1011, 72.3%).

**Clinical Samples collected at**	**Screening n (%)**	**Random assignment n (%)**	**Clinical performance testing completed n (%)**	**Samples included in analysis Set n (%)**
Konkuk University Hospital	388 (27.7)	280 (24.7)	280 (24.7)	280 (24.7)
Seoul National University Hospital	1,011 (72.3)	852 (75.3)	852 (75.3)	852 (75.3)
**Total**	**1,399 (100.0)**	**1,132 (100.0)**	**1,132 (100.0)**	**1,132 (100.0)**

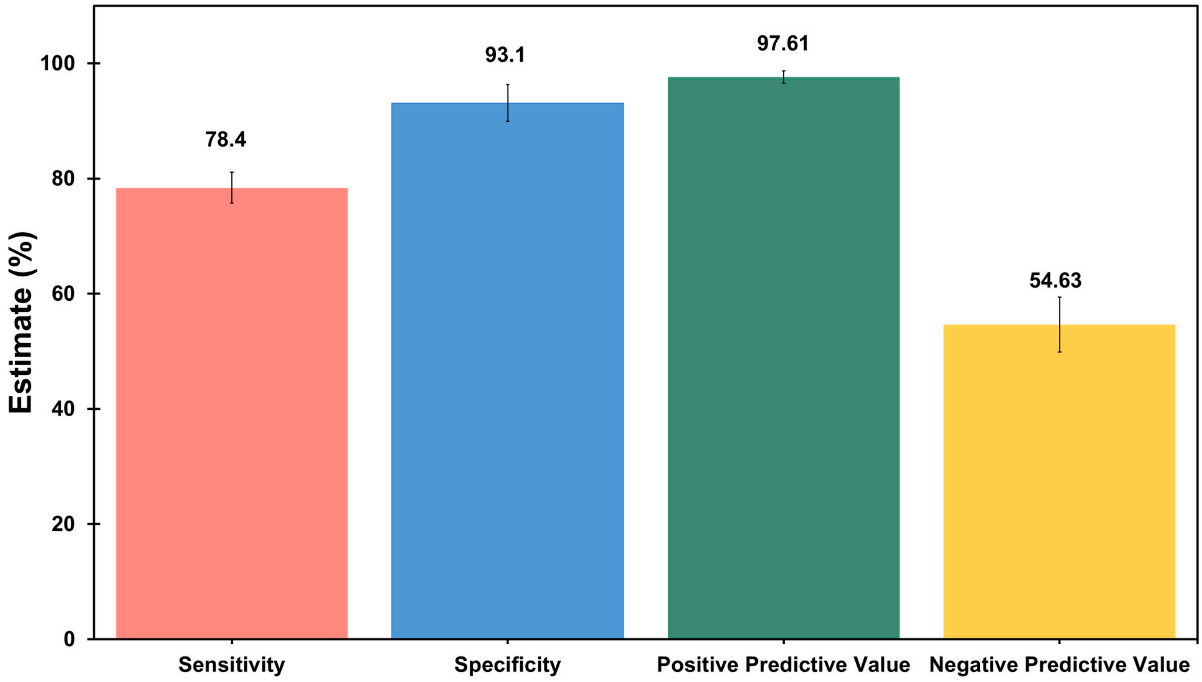

Figure S1. Sensitivity, Specificity, PPV and NPV of 9G test™ Cancer/Lung test for identification of risk of lung cancer.

Table S2. Gender-specific sensitivity and specificity of 9G test™ Cancer/Lung test for identification of risk of lung cancer.

Gender	Cancer (N = 885)	Benign (N = 247)
**Men**	**566**	**137**
High-risk of lung cancer (Index > 3.5)	449 (79.3%)	9 (6.6%)
Low-risk of lung cancer (Index ≤ 3.5)	117 (20.7%)	128 (93.4%)
Sensitivity		
Estimates (%)	79.3	-
(95% CI)	(76.6, 82.7)	-
Specificity		
Estimates (%)	93.4	-
(95% CI)	(89.3, 97.6)	-
**Women**	**319**	**110**
High-risk of lung cancer (Index > 3.5)	245 (76.8%)	8 (7.3%)
Low-risk of lung cancer (Index ≤ 3.5)	74 (23.2%)	102 (92.7%)
Sensitivity		
Estimates (%)	76.8	-
(95% CI)	(72.2, 81.4)	-
Specificity		
Estimates (%)	92.7	-
(95% CI)	(87.9, 97.6)	-

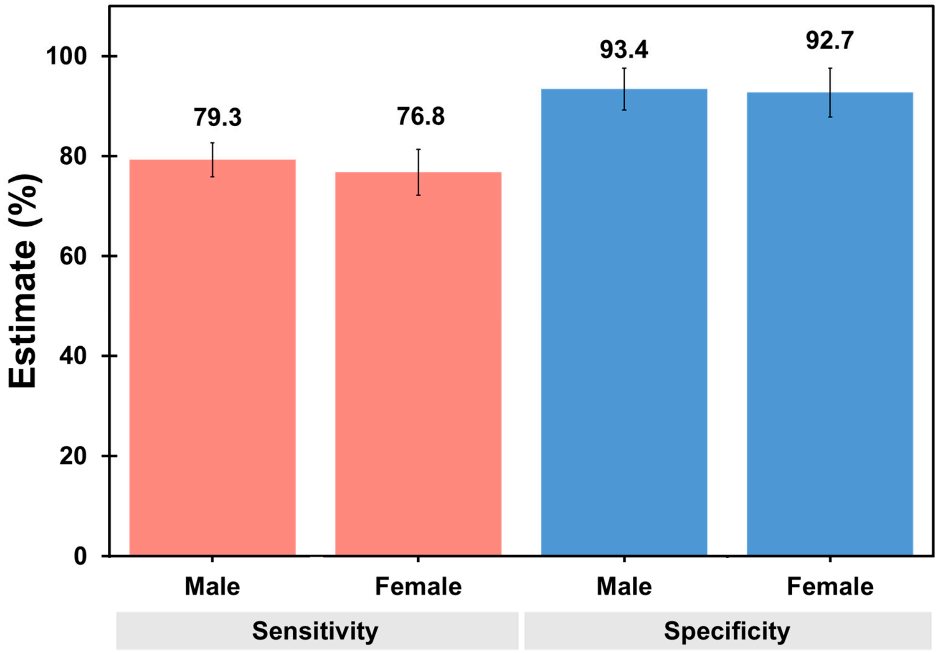

Figure S2. Gender specific sensitivity and specificity of 9G test™ Cancer/Lung test for identification of risk of lung cancer.

Table S3. Sensitivity and specificity of the 9G test™ Cancer/Lung test for identifying the risk of lung cancer in various age groups.

**Age groups, Years**	**Cancer (N=885)**	**Benign (N=247)**
**50 – 59**	**153**	**75**
High-risk of lung cancer (Index > 3.5)	120 (78.4%)	4 (5.3%)
Low-risk of lung cancer (Index ≤ 3.5)	33 (21.6%)	71 (94.7%)
Sensitivity		
Estimates (%)	78.4	-
(95% CI)	(71.9, 85.5)	-
Specificity		
Estimates (%)	94.7	-
(95% CI)	(89.6, 99.8)	-
**60 – 69**	**380**	**72**
High-risk of lung cancer (Index > 3.5)	300 (79.0%)	5 (6.9%)
Low-risk of lung cancer (Index ≤ 3.5)	80 (21.0%)	67 (93.1%)
Sensitivity		
Estimates (%)	79.0	-
(95% CI)	(74.9, 83.1)	-
Specificity		
Estimates (%)	93.1	-
(95% CI)	(87.2, 98.9)	-
**70 – 79**	**352**	**100**
High-risk of lung cancer (Index > 3.5)	274 (77.8%)	8 (8.0%)
Low-risk of lung cancer (Index ≤ 3.5)	78 (22.2%)	92 (92.0%)
Sensitivity		
Estimates (%)	77.8	-
(95% CI)	(73.5, 82.2)	-
Specificity		
Estimates (%)	92.0	-
(95% CI)	(86.7, 97.3)	-

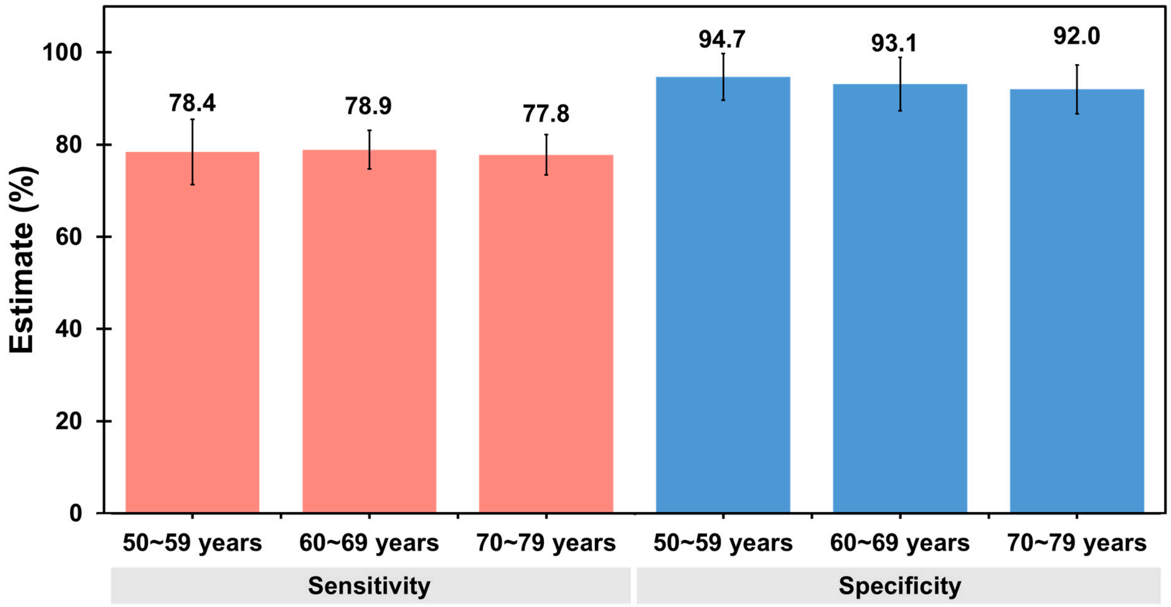

Figure S3. Age-group specific sensitivity and specificity of 9G test™ Cancer/Lung test for identification of risk of lung cancer.

Table S4. Smoking-history specific sensitivity and specificity of 9G test™ Cancer/Lung test for identification of risk of lung cancer.

**Smoking history**	**Cancer (N = 885)**	**Benign (N = 247)**
Smoker	**556**	**125**
High-risk of lung cancer (Index > 3.5)	438 (78.8%)	7 (5.6%)
Low-risk of lung cancer (Index ≤ 3.5)	118 (21.2%)	118 (94.4%)
Sensitivity		
Estimates (%)	78.8	-
(95% CI)	(75.4, 82.2)	-
Specificity		
Estimates (%)	94.4	-
(95% CI)	(90.4, 98.4)	-
Never smoker	**329**	**122**
High-risk of lung cancer (Index > 3.5)	256 (77.8%)	10 (8.2%)
Low-risk of lung cancer (Index ≤ 3.5)	73 (22.2%)	112 (91.8%)
Sensitivity		
Estimates (%)	77.8	-
(95% CI)	(73.3, 82.3)	-
Specificity		
Estimates (%)	91.8	-
(95% CI)	(86.9, 96.7)	-

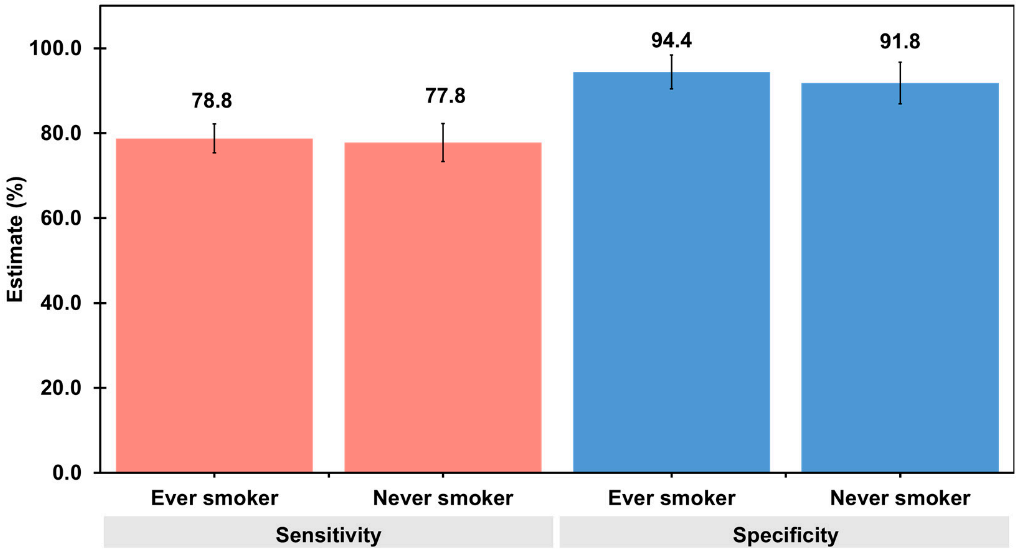

Figure S4. Smoking-history specific sensitivity and specificity of 9G test™ Cancer/Lung test for identification of risk of lung cancer.

Table S5. Tumor tissue histology based sensitivity and specificity of 9G test™ Cancer/Lung test for identification of risk of lung cancer.

**Cancer Type**	**Cancer (N = 885)**	**Benign (N = 247)**
Non-Small Cell Lung Cancer (NSCLC)	**757**	**247**
High-risk of lung cancer (Index > 3.5)	591 (78.1%)	17 (6.9%)
Low-risk of lung cancer (Index ≤ 3.5)	166 (21.9%)	230 (93.1%)
Sensitivity		
Estimates (%)	78.1	-
(95% CI)	(75.1, 81.0)	-
Specificity		
Estimates (%)	93.1	-
(95% CI)	(90.0, 96.3)	-
Small Cell Lung Cancer (SCLC)	**128**	**247**
High-risk of lung cancer (Index > 3.5)	103 (80.5%)	17 (6.9%)
Low-risk of lung cancer (Index ≤ 3.5)	25 (19.5%)	230 (93.1%)
Sensitivity		
Estimates (%)	80.5	-
(95% CI)	(73.6, 87.3)	-
Specificity		
Estimates (%)	93.1	-
(95% CI)	(90.0, 96.3)	-

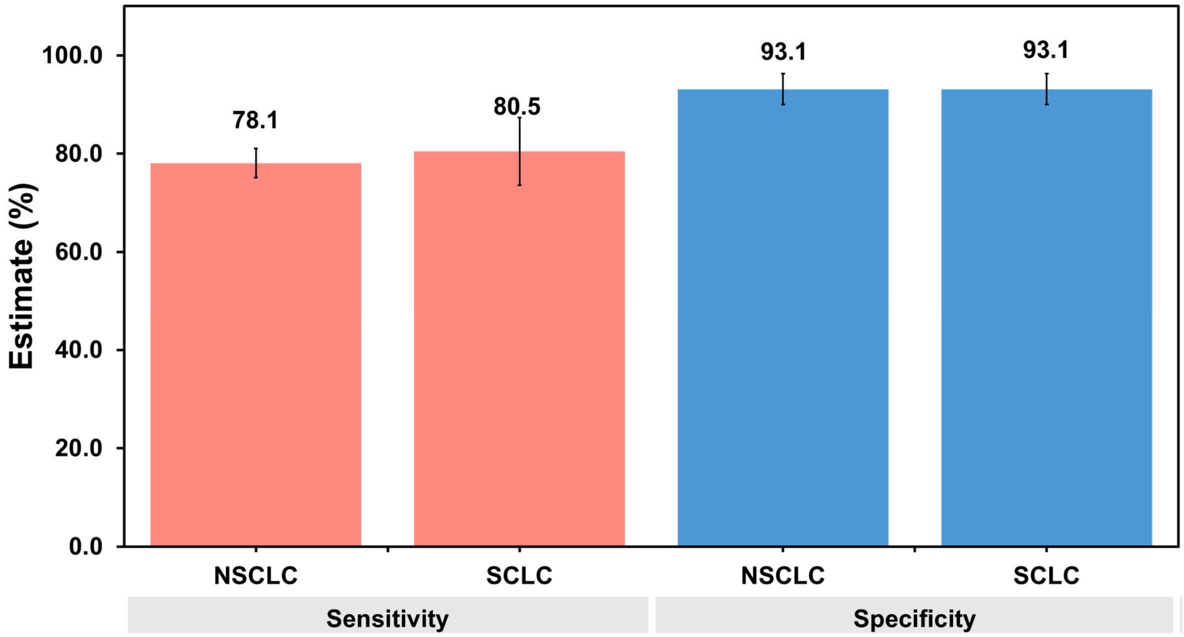

Figure S5. Tumor tissue histology based sensitivity and specificity of 9G test™ Cancer/Lung test for identification of risk of lung cancer.

Table S6. Lung cancer stage-specific sensitivity and specificity of 9G test™ Cancer/Lung test for identification of risk of lung cancer.

Cancer Stages	Cancer (N = 885)	Benign (N = 247)
**Stage I**	**408**	**247**
High-risk of lung cancer (Index > 3.5)	329 (80.6%)	17 (6.9%)
Low-risk of lung cancer (Index ≤ 3.5)	79 (19.4%)	230 (93.1%)
Sensitivity		
Estimates (%)	80.6	-
(95% CI)	(76.8, 84.5)	-
Specificity		
Estimates (%)	93.1	-
(95% CI)	(90.0, 96.3)	-
**Stage II**	**142**	**247**
High-risk of lung cancer (Index > 3.5)	104 (73.2%)	17 (6.9%)
Low-risk of lung cancer (Index ≤ 3.5)	38 (26.8%)	230 (93.1%)
Sensitivity		
Estimates (%)	73.2	-
(95% CI)	(66.0, 80.5)	-
Specificity		
Estimates (%)	93.1	-
(95% CI)	(90.0, 96.3)	-
**Stage III**	**146**	**247**
High-risk of lung cancer (Index > 3.5)	108 (74.0%)	17 (6.9%)
Low-risk of lung cancer (Index ≤ 3.5)	38 (26.0%)	230 (93.1%)
Sensitivity		
Estimates (%)	74.0	-
(95% CI)	(66.9, 81.1)	-
Specificity		
Estimates (%)	93.1	-
(95% CI)	(90.0, 96.3)	-
**Stage IV**	**61**	**247**
High-risk of lung cancer (Index > 3.5)	50 (82.0%)	17 (6.9%)
Low-risk of lung cancer (Index ≤ 3.5)	11(18.0%)	230 (93.1%)
Sensitivity		
Estimates (%)	82.0	-
(95% CI)	(72.3, 91.6)	-
Specificity		
Estimates (%)	93.1	-
(95% CI)	(90.0, 96.3)	-
**Limited Disease (LD)**	**57**	**247**
High-risk of lung cancer (Index > 3.5)	47 (82.5%)	17 (6.9%)
Low-risk of lung cancer (Index ≤ 3.5)	10 (17.5%)	230 (93.1%)
Sensitivity		
Estimates (%)	82.5	-
(95% CI)	(72.6, 92.3)	-

Cancer Stages	Cancer (N = 885)	Benign (N = 247)
Specificity		
Estimates (%)	93.1	-
(95% CI)	(90.0, 96.3)	-
**Extensive Disease (ED)**	**71**	**247**
High-risk of lung cancer (Index > 3.5)	56 (78.9%)	17 (6.9%)
Low-risk of lung cancer (Index ≤ 3.5)	15 (21.1%)	230 (93.1%)
Sensitivity		
Estimates (%)	78.8	-
(95% CI)	(69.4, 88.4)	-
Specificity		
Estimates (%)	93.1	-
(95% CI)	(90.0, 96.3)	-

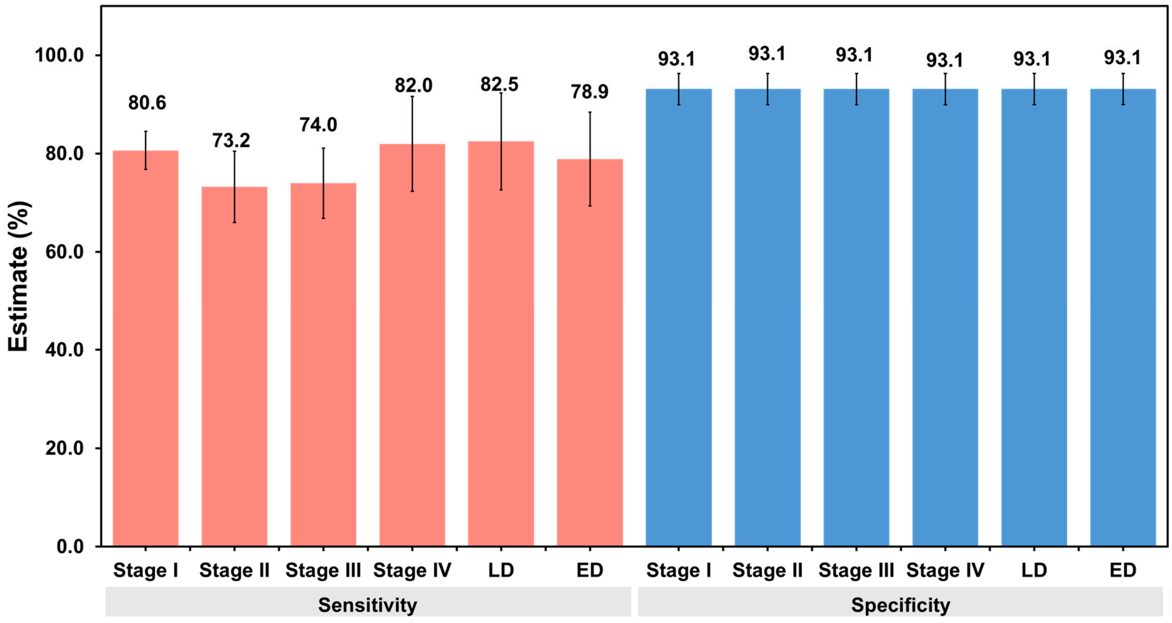

Figure S6. Lung cancer stage-specific sensitivity and specificity of 9G test™ Cancer/Lung test for identification of risk of lung cancer.
